# Supplementary material for: Network Pharmacology, Molecular Docking and Molecular Dynamics to Explore the Potential Immunomodulatory Mechanisms of Deer Antler
Source: Int J Mol Sci. 2023 Jun 20;24(12):10370. doi: 10.3390/ijms241210370 (PMC10299714; doi:10.3390/ijms241210370)
Supplement: Supplementary file 1 [file ijms-24-10370-s001.zip › Supplemental Figure S1.pptx]

## Slide 1
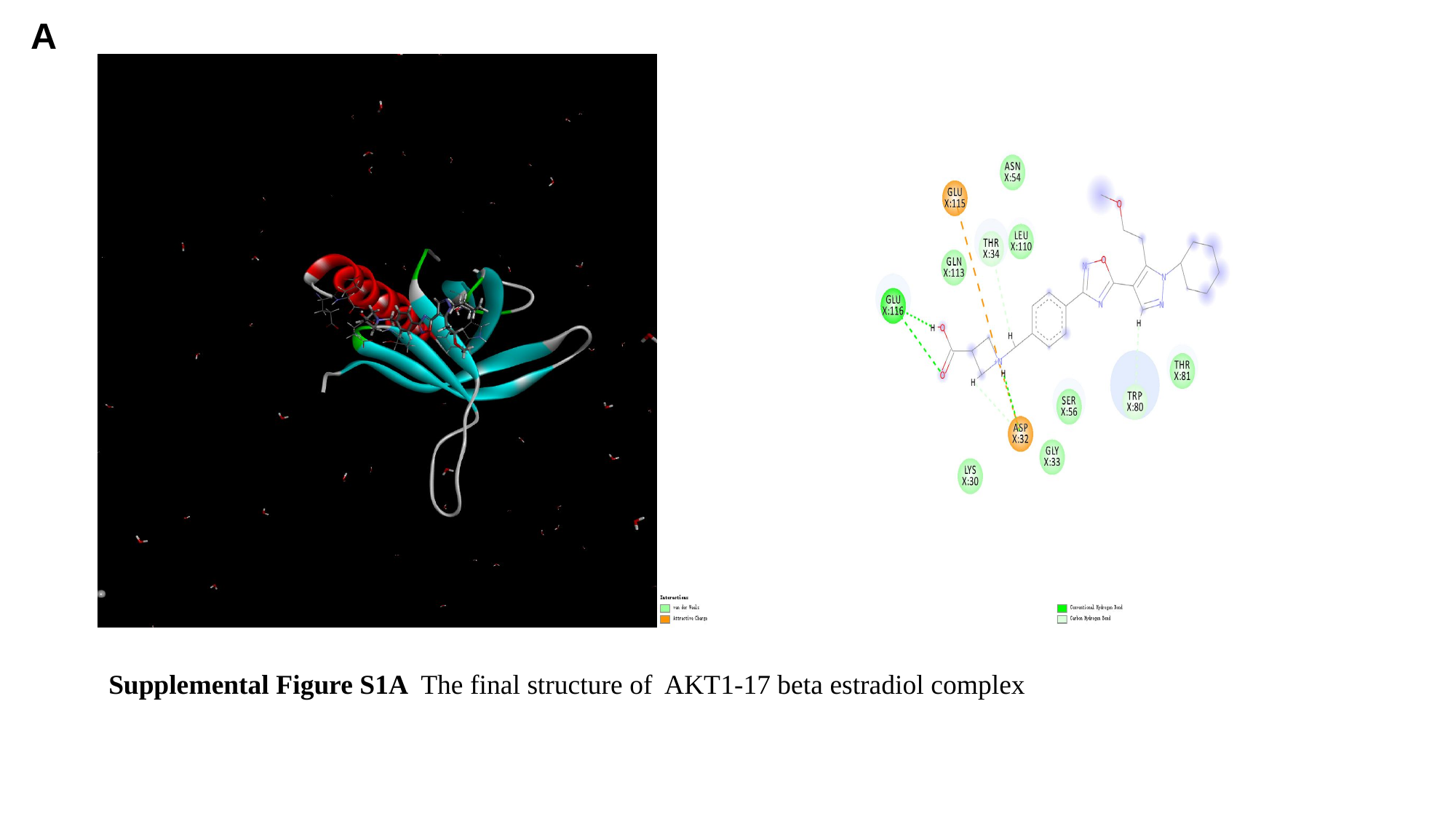

A
Supplemental Figure S1A The final structure of AKT1-17 beta estradiol complex

## Slide 2
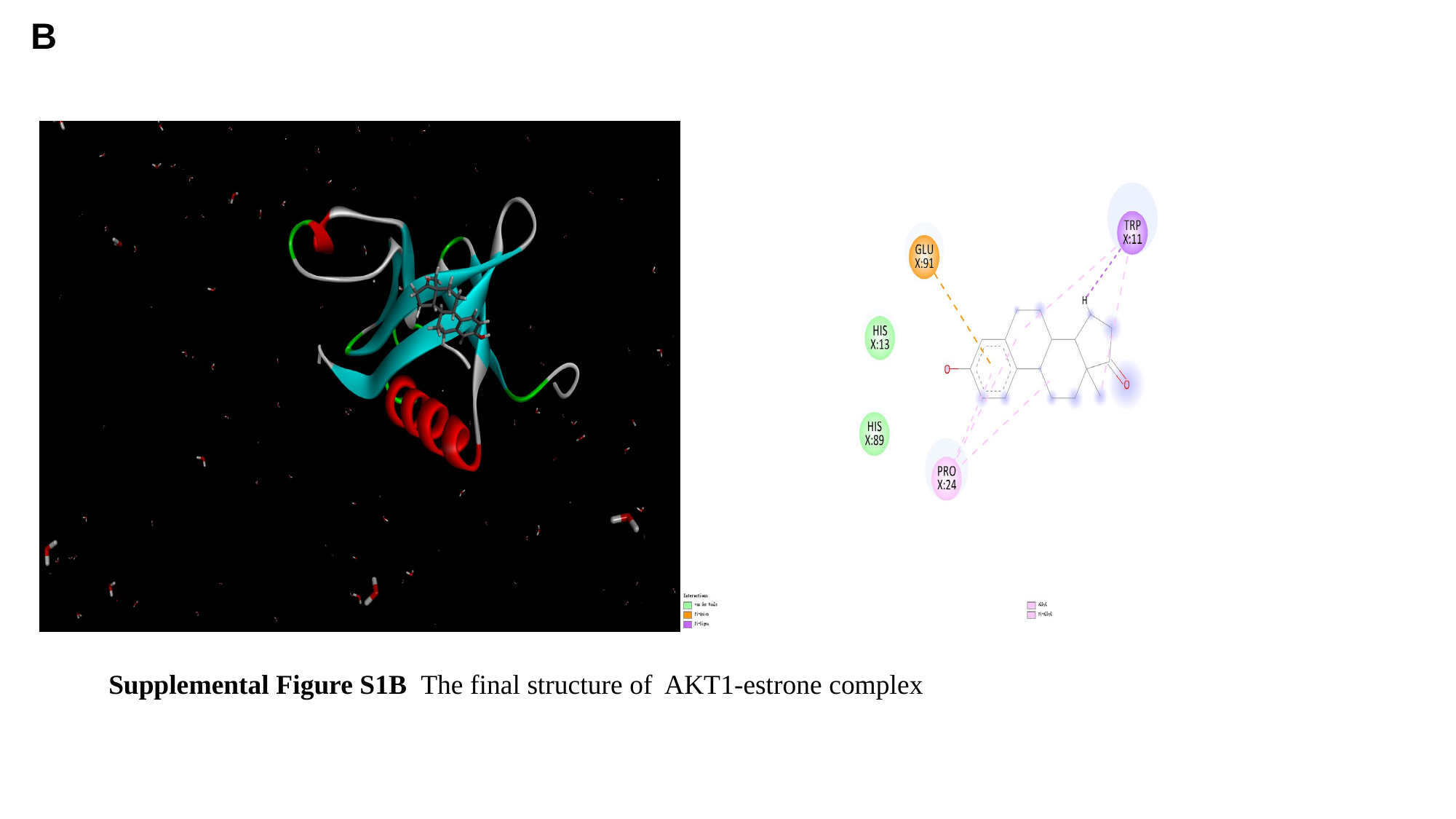

B
Supplemental Figure S1B The final structure of AKT1-estrone complex

## Slide 3
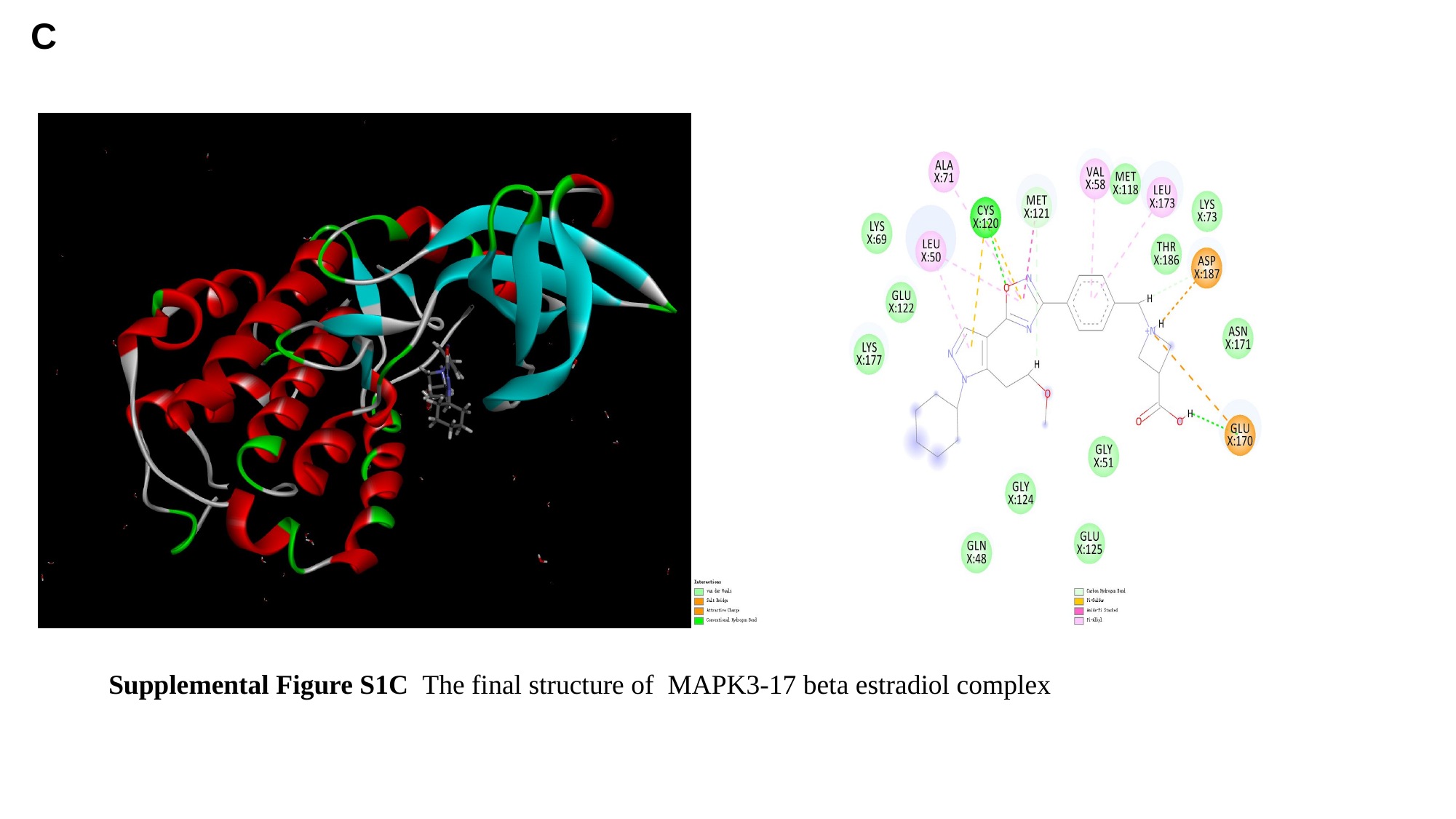

C
Supplemental Figure S1C The final structure of MAPK3-17 beta estradiol complex

## Slide 4
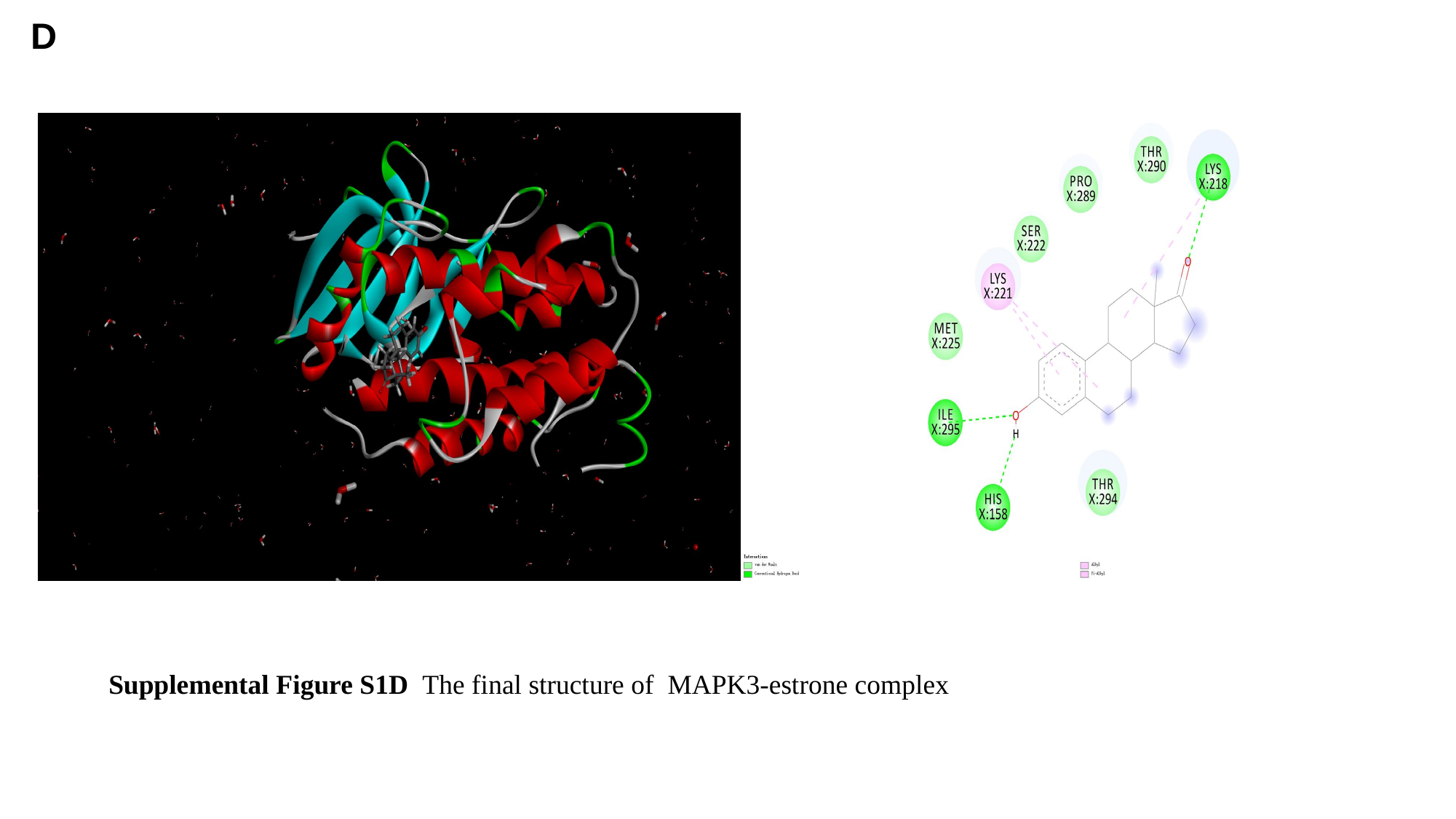

D
Supplemental Figure S1D The final structure of MAPK3-estrone complex
