# Supplementary material for: Network Pharmacology, Molecular Docking and Molecular Dynamics to Explore the Potential Immunomodulatory Mechanisms of Deer Antler
Source: Int J Mol Sci. 2023 Jun 20;24(12):10370. doi: 10.3390/ijms241210370 (PMC10299714; doi:10.3390/ijms241210370)
Supplement: Supplementary file 1 [file ijms-24-10370-s001.zip › Supplemental Figure S3.pptx]

## Slide 1
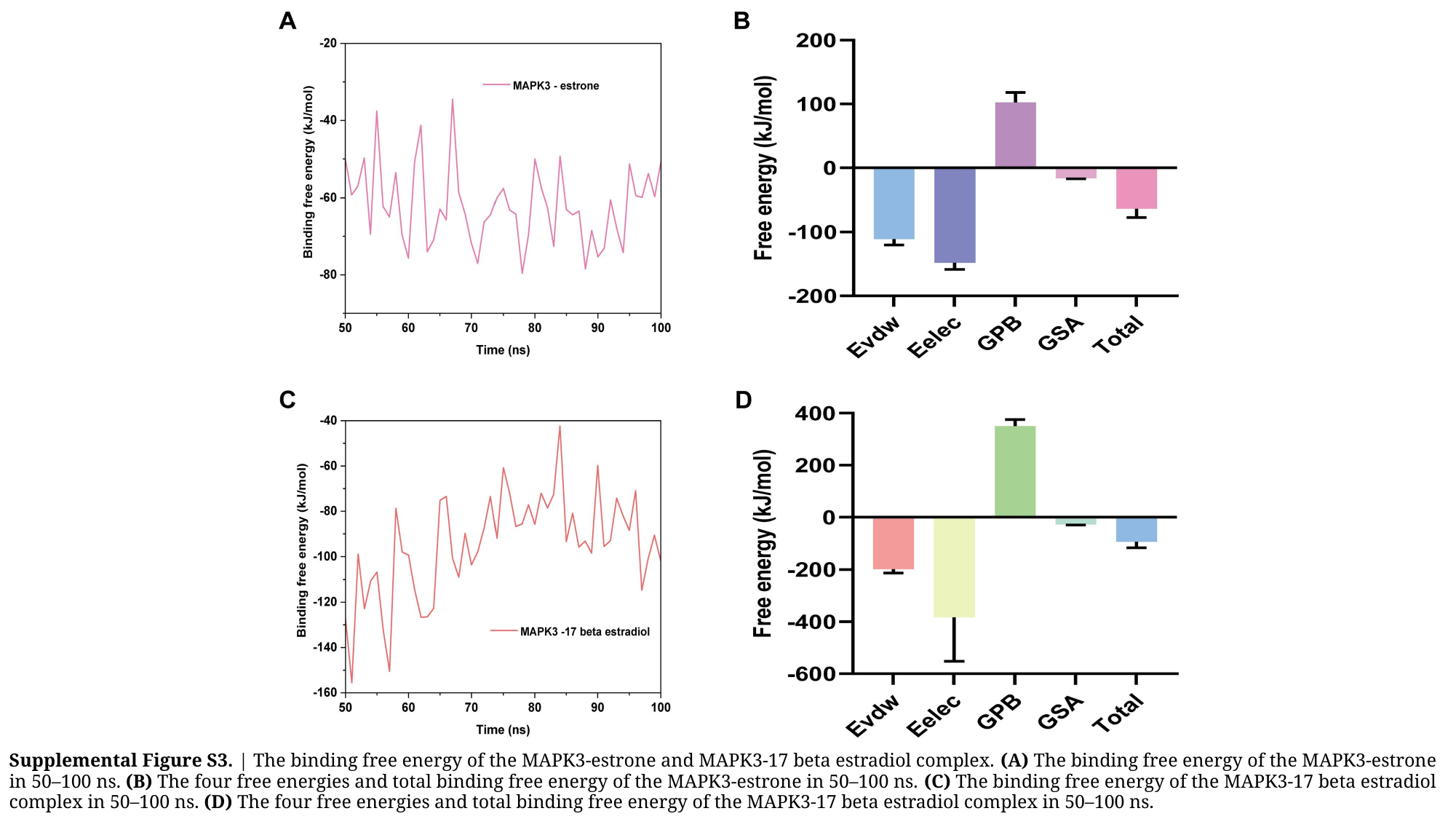

Supplemental Figure S3. | The binding free energy of the MAPK3-estrone and MAPK3-17 beta estradiol complex. (A) The binding free energy of the MAPK3-estrone in 50–100 ns. (B) The four free energies and total binding free energy of the MAPK3-estrone in 50–100 ns. (C) The binding free energy of the MAPK3-17 beta estradiol complex in 50–100 ns. (D) The four free energies and total binding free energy of the MAPK3-17 beta estradiol complex in 50–100 ns.
